# *Fasciola hepatica* expresses multiple α- and β-tubulin isotypes

**DOI:** 10.1016/j.molbiopara.2008.02.001

**Published:** 2008-05

**Authors:** Louise A. Ryan, Elizabeth Hoey, Alan Trudgett, Ian Fairweather, Marc Fuchs, Mark W. Robinson, Emma Chambers, David J. Timson, Eimear Ryan, Theresa Feltwell, Al Ivens, Geoffrey Bentley, David Johnston

**Affiliations:** a School of Biological Sciences, Queen's University of Belfast, 97 Lisburn Road, Belfast BT9 7BL, United Kingdom; b School of Medical Sciences, University of Aberdeen, Aberdeen AB25 2ZD, United Kingdom; c Wellcome Trust Sanger Institute, Hinxton, Cambridge CB10 1SA, United Kingdom; d School of Biology, University of Leeds, Leeds LS2 9JT, United Kingdom; e The Natural History Museum, London SW7 5BD, United Kingdom

**Keywords:** *Fasciola hepatica*, Trematode, Tubulin, Drug resistance, Isotypes

## Abstract

We have identified five α-tubulin and six β-tubulin isotypes that are expressed in adult *Fasciola hepatica*. Amino acid sequence identities ranged between 72 and 95% for fluke α-tubulin and between 65 and 97% for β-tubulin isotypes. Nucleotide sequence identity ranged between 68–77% and 62–80%, respectively, for their coding sequences. Phylogenetic analysis indicated that two of the α-tubulins and two of the β-tubulins were distinctly divergent from the other trematode and nematode tubulin sequences described in this study, whereas the other isotypes segregated within the trematode clades. With regard to the proposed benzimidazole binding site on β-tubulin, three of the fluke isotypes had tyrosine at position 200 of β-tubulin, two had phenylalanine and one had leucine. All had phenylalanine at position 167 and glutamic acid at position 198. When isotype RT-PCR fragment sequences were compared between six individual flukes from the susceptible Cullompton isolate and from seven individual flukes from the two resistant isolates, Sligo and Oberon, these residues were conserved.

Microtubules are essential and dynamic cytoskeletal components of all eukaryotic cells. They are polymers of the protein tubulin, a heterodimer of two subunits, α- and β-tubulin, that are highly conserved across species. These subunits are structurally similar and share approximately 40% amino acid sequence identity [1]. Tubulin is the target of many potential or effective parasite therapeutic drugs. The subtle differences between the tubulins of the host and parasite are sufficient to provide selective toxicity for some agents. For example, certain dinitroanilines bind α-tubulins of protozoan parasites but not the host, making them good drug candidates [2] and certain benzimidazoles (BZ) bind to β-tubulins of nematodes [3].

The drug of choice for the treatment of *Fasciola hepatica*, the causative agent of fasciolosis, is triclabendazole (TCBZ), a BZ-derivative. Although TCBZ is not a classical BZ, morphological studies have indicated that there is severe disruption of microtubule-based processes in the tegument, in spermatogenic cells of the testis and in vitelline cells following treatment of susceptible adult fluke [4], suggesting that tubulin is also its target. Unfortunately, resistance to this drug is emerging in various locations worldwide [4,5]. This is alarming since the incidence of fasciolosis is increasing in regions of the UK due to the advent of warmer and wetter climatic conditions, which favour the proliferation of the intermediate host, the snail *Galba truncatula* [6].

In *Haemonchus contortus*, BZ resistance results from selection of parasites that have a β-tubulin isotype 1 subunit with certain single amino acid differences from those occurring in susceptible isolates. The relevant differences occur at the BZ binding site [7] and are either at position 167 where phenylalanine is replaced by tyrosine or histidine [8], at position 198 where glutamic acid is replaced by alanine [9], or at position 200 where phenylalanine is substituted by tyrosine [3].

To date, few studies have examined the tubulin isotypes of the liver fluke, *F. hepatica*. Robinson et al. [10,11] have described one β-tubulin isotype that is expressed in adult fluke and have reported that this has an identical sequence in fluke from an isolate that has been reported to be TCBZ-susceptible [11] and an isolate that has been documented to be TCBZ-resistant [11,12]. In this study, we wished to investigate if adult *F. hepatica* express multiple α- and β-tubulin isotypes, as is the case for most species. We initially used PCR amplification of cDNAs from the Cullompton TCBZ-susceptible isolate, which is maintained in our laboratory [11]. Unless otherwise stated, standard PCR cycling conditions were employed. The PCR primers used are given in Table 1. Numbering refers to the 5′ position of each primer on the coding sequence. This revealed that the *Fasciola* genome encodes at least four α-tubulin isotypes, *F.hep*-α-tub1 to *F.hep*-α-tub4, and three β-tubulin isotypes, *F.hep*-β-tub1 to *F.hep*-β-tub3. We also performed database searches with all of the 5000 individual adult *F. hepatica* expressed sequence tagged (EST) reads from a pilot gene discovery project (available from ftp://ftp.sanger.ac.uk/pub4/pathogens/Fasciola/hepatica/ESTs/). These sequences were not annotated. The five flukes used to create the EST library came from an abattoir in Co. Down, Northern Ireland. In this EST database, *F.hep*-β-tub2, *F.hep*-β-tub3 and *F.hep*-α-tub4 coding sequences were not represented but readings representing a fifth α-tubulin isotype (*F.hep*-α-tub5) and three new β-tubulin isotypes were found (*F.hep*-β-tub4 to *F.hep*-β-tub6). The identified tubulin inserts in EST clones were completely sequenced and merged with our preliminary data. When necessary, sequences were completed using rapid amplification of cDNA ends experiments (RACE Smart kit; Clontech) using the supplier's recommendations for primer design and PCR conditions. More detailed experimental methods are given in the legends to Fig. 1A and B. All overlaps used in the compilations showed greater than 99% identity within each isotype. Observed variations occurred mostly at the third base of codons and the majority did not give rise to amino acid changes and probably represent allelic variation. Subsequent PCR experiments, using Cullompton cDNA, allowed the full-length coding regions of all isotypes to be amplified, cloned and sequenced from this isolate. All isotypes are expressed in individual fluke. These Cullompton sequences, used in the subsequent analysis, have been submitted to the EMBL database and can be accessed using the GenBank accession numbers AM933580–AM933584 for the five α-tubulins and AM933585–AM933590 for the six β-tubulins.

All of the encoded protein subunits have the tubulin signature sequence, GGGTGSG, which binds to the α- and β-phosphates of the bound GTP/GDP nucleotides [1]. The isotype amino acid sequences and the sequence identities of their coding sequences are given in Fig. 2. Although α-tubulins 1 and 2 sequences are very similar, with 95% amino acid identity, their coding regions are more divergent, having only 76% nucleotide identity. This indicates that they are distinct gene products. The β-tubulin isotypes 1–3 are also very similar (97% amino acid identity), but have between 73 and 80% nucleotide identity when their coding sequences are considered. Moreover, following analysis of genomic PCR fragments, generated using primers β-tub 295f and β-tub 1230r [13], we have shown that the β-tubulin isotype 2 gene contains an intron of 78 nucleotides at coding position 525 in genomic DNA whereas β-tubulin3 has an intron of 49 nucleotides at the same position. We have also confirmed that β-tubulin isotype 1 does not contain an intron at this positon. The other α- and β-isotypes show substantial variation between isotypes at both the amino acid (α-tubulins 72–82%; β-tubulins 65–93%) and nucleotide levels (α-tubulin coding regions 68–73%; β-tubulin coding regions 62–80%).

A Clustal W alignment of *Fasciola* α-tubulin isotypes is shown in Fig. 1A. The secondary structure established for the refined porcine αβ-tubulin model [14] is indicated above the alignment. The major sequence variation between the isotypes occurs at their acidic C-terminal ends. This region contributes to the isotype-defining regions in other species [15]. In addition, two other regions show substantial inter-isotypic variability: firstly, amino acid positions 36–55, which correspond to the N-terminal loop connecting helix 1 and β-strand 2, a region reported to have poor density in the αβ-tubulin model data [14]; secondly, amino acid positions 325–342 corresponding to helix 10 in the three-dimensional structure, which plays a part in both longitudinal interactions within protofilaments and in lateral contacts between protofilaments [16], and so may influence microtubule stability. Three of the isotypes, *F.hep*-α-tub1, 2 and 3, have a lysine at position 40 and potentially can be acetylated, a modification that is associated with stable microtubules [17]. Isotypes *F.hep*-α-tub4 and 5 have an asparagine at this position. However, *F.hep*-α-tub4 has a lysine at position 39. Since there is a one amino acid deletion in the loop in which this occurs, compared to isotypes 1–3, this lysine could be in an equivalent position to be acetylated. Isotypes 1–4 terminate with Glu-Tyr and thus have the potential to participate in a tyrosination/detyrosination cycle. Detyrosinated tubulins are associated with stable microtubules such as those found in axonemes [17]. The site for glutamylation at residue 445 is absent in isotypes 4 and 5, making it unlikely that they could interact with the fluke equivalent of kinesin KIF1 in the transport of synaptic vesicles in neurons [18]. The *F.hep*-α-tub5 isotype has an N-terminal extension of five amino acids compared to the other α-tubulins, which is similar to that occurring in the isotype 9 of *Caenorhabditis elegans* (GenBank accession no. Q20221).

The most notable feature portrayed by the β-tubulin alignment (Fig. 1B) is the absence of a large part of the variable C-terminal acidic tail in isotypes 5 and 6 compared to the other β-tubulins. From structural studies, the tail has been shown to be situated at the outside of the microtubule and is thought to interact with microtubule-associated proteins [16]. Isotypes 1–3 have the axonemal signal sequence, EGEF followed by three acidic amino acids [17], in their C-terminal regions. Of interest are the residues involved in BZ resistance in nematodes and which have been reported to be associated with the BZ binding site [19]. Phenylalanine is present in all *Fasciol*a isotypes at position 167. At position 198, a glutamic acid residue is present in all isotypes. At position 200, isotypes 1–3 have tyrosine, isotypes 4 and 6 have phenylalanine and isotype 5 has leucine. These amino acids were encoded at these positions in RT-PCR fragments generated for all of the isotypes from five Cullompton TCBZ-susceptible isolate fluke, from two fluke from the Sligo TCBZ-resistant isolate [11,12] and from five fluke from the Oberon TCBZ-resistant isolate [20]. These resistant flukes survived TCBZ-treatment and drug efficacies have been reported to be less than 5% 4 weeks following treatment *in vivo* [12,20] compared to over 96% for the Cullompton isolate [21]*.* In sequencing chromatograms, single peaks were recorded at the codon positions, indicating that the flukes were homozygous at these sites.

Although morphological studies have indicated that microtubules are more disrupted in Cullompton TCBZ-susceptible fluke than in the Sligo TCBZ-resistant isolate following treatment with the drug [11], we do not know for certain that β-tubulin is the target protein, and, if not, that mutations in the true target protein are responsible for resistance in these isolates. Other processes have been implicated in the resistance mechanism in the Sligo isolate. HPLC analysis has revealed that flukes from this isolate accumulate less TCBZ and triclabendazole suphoxide than the Cullompton-susceptible isolate following *in vitro* drug treatment [22]. This could be reversed by incubating with ivermectin suggesting that an ABC transporter protein, such as *P*-glycoprotein, is involved. In addition the Sligo-resistant isolate has been shown to have a higher rate of TCBZ metabolism than the susceptible isolate following drug treatment [22].

The number of tubulin isotypes can vary between organisms. The protozoa, *Trypanosoma cruzi* has one α- and one β-tubulin protein sequence [23] whereas the nematode *C. elegans* has nine α- and six β-tubulin isotypes [24]. This is the first extensive study documenting both α- and β-tubulin isotypes from a platyhelminth although analysis of the available *Schistosoma mansoni* genome and EST databanks suggest that this trematode encodes at least five α- and six β-tubulin isotypes. Available full-length tubulin trematode sequences, together with selected nematode, cestode and protozoan parasite tubulin isotypes, were used in phylogenetic analyses using the Neighbor–Joining method [25] and the Mega 4.0 program [26]. In the α-tubulin tree (Fig. 2A), *F.hep*-α-tub1, 2 and 3 clustered with the platyhelminth clade. This clade is separate from that containing the nematode sequences and from that containing the protozoan parasite sequences. The isotypes *F.hep*-α-tub4 and 5 occur on separate branches, distal from the other *Fasciola* sequences. In the β-tubulin tree, *F.hep*-β-tub1–4 group with the other trematode sequences and with isotype 2 of *E. multilocularis*, whilst the other two fluke isotypes, *F.hep*-β-tub5 and 6, again occur as distinct branches indicating that they are very different. Additional *S. mansoni* full-length sequences to those used in the above analysis were obtained by BLAST searching of the *S. mansoni* genome and EST project sequences using the GeneDB programme [27] hosted at the Sanger Centre. These were contig sequences and the majority had temporary identification numbers. Phylogenetic analyses performed with these sequences included showed that they grouped in the platyhelminth clade (data not shown). Interestingly, the nematode sequences in both the α- and β-trees form clades distinct from those containing the trematode sequences, suggesting structural differences that may underlie their differing susceptibilities to certain benzimidazoles.

The major separation of two α-isotypes and the two β-isotypes from the others in the platyhelminth clade could suggest that they have resulted from early duplication events or have evolved to fulfill specialized functions within the fluke. Another possibility (originally suggested by Burland et al. [28] to explain a variant β-tubulin of the mitotic spindle of *Physarum polycephalum*) is that differing isotypes can arise from neutral drift, because if used exclusively for one function, they will have less changes in regions associated with that function whilst being more open to changes in regions associated only with other cellular processes. BLAST searching of the Schistosome databanks with full-length *Fasciola* sequences and with 200 bp fragments overlapping by 100 bp, to try to ensure that small ESTs were investigated, suggested that there are no tubulins similar to *F.hep*-α-tub4 and 5 or *F.hep*-β-tub5 and 6 encoded in their genomes.

For a number of the species included in the phylogenetic analyses there are numerous partial tubulin sequences that are in the databases, mainly as a result of EST and genome projects. Apart from the schistosome data, the nematode *H. contortus* has been shown to contain two more β-tubulin genes, in addition to the isotypes 1 and 2 genes previously documented when its genome sequence was examined [29]. Thus, a fuller *Fasciola* genome and/or EST project may reveal other members of these multi-gene families in this parasite.

In summary, we have shown that the *Fasciola* genome encodes at least five α- and six β-tubulin isotypes that are expressed at the adult stage of the life cycle. Of the sequences identified, three α- and four β-isotypes group with the other trematode tubulins in phylogenetic analysis, whereas the others are more diverse. The determination of these sequences should allow their *in vitro* expression, which in turn may allow investigation as to which of these isotypes, if any, TCBZ binds. The data will also form the basis of the full-scale characterization of the microtubular system in the fluke and may enable other specific fasciolides to be designed.

## Figures and Tables

**Fig. 1:**
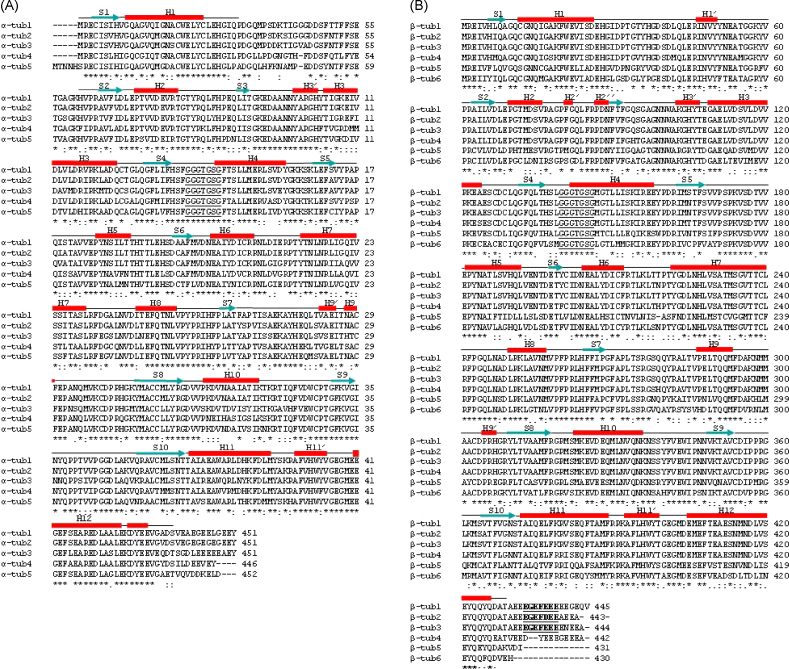
Clustal W sequence alignments of Cullompton triclabendazole-susceptible isolate (A) α-tubulin isotypes, *F.hep*-α-tub1, 2, 3, 4 and 5 and (B) β-tubulin isotypes, *F.hep*-β-tub1, 2, 3, 4, 5 and 6 predicted from their coding sequences. The tubulin signal sequence motifs are underlined and the axonemal signal motif is in bold in the β-tubulin alignments. The secondary structures of porcine α- and β-tubulin are shown above the alignments (adapted from [14]). Gaps represent regions of low density in the crystal structure data. H: helix, S: β-strand. Initial full-length sequence was obtained using data from PCR-based experiments using Cullompton isolate cDNA and from adult *Fasciola* EST clones. Primer sequences are given in Table 1. When isotype coding sequences were complete, primers were developed that allowed full-length coding sequences to be cloned and sequenced from Cullompton isolate cDNA. These sequences are presented. The initial sequences were established as follows. (A) α-tubulin isotypes. *F.hep*-α-tub1: a cloned PCR fragment generated using degenerate primers α-tub 44 and α-tub 637 (Table 1) from *Fasciola* cDNA gave 600 bp of the coding sequence of *F.hep*-α-tub1. The 5′ end of the coding sequence was obtained from a RACE product using primer A1 490r 5′RACE. The EST clone 36h02 contained the 3′ end of this isotype. *F.hep*-α-tub2: the complete coding sequence was determined from EST clones 51d06 and 52e12. *F.hep*-α-tub3: the complete coding sequence was determined from EST clone 42e05. *F.hep*-α-tub4: an PCR fragment, generated using primers α-tub 397 and α-tub 1160, gave 760 bp of the *F.hep*-α-tub4 coding sequence and was completed using 5′ and 3′RACE products generated using primers A4 718r 5′RACE and A4 543f 3′RACE, respectively. *F.hep*-α-tub5: plasmid inserts in clones 09c01 and 39b11 contained the complete-coding sequence. (B) β-tubulin isotypes. *F.hep*-β-tub1: the EST clone 06g07 contained the 3′ end of the coding region of the coding sequence and completed the previously reported sequence of *F.hep*-β-tub1 [11]. *F.hep*-β-tub2 and 3: cloned fragments (approximately 1 kb), generated using degenerate primers β-tub 295f and β-tub 1230r and conditions given in [13], yielded two separate partial coding sequences for these isotypes. They were completed from 5′ and 3′RACE cDNA fragments generated using primers B2 737r 5′RACE and 712F 3′RACE for *F.hep*-β-tub2 and primers B3 519r 5′RACE and B3 361f 3′RACE for *F.hep*-β-tub3. *F.hep*-β-tub4: the plasmid insert of EST clone 28d07 contained from coding sequence nucleotides 1097 to the poly A tract. Further sequence was obtained following amplification of a cDNA fragment using primers β-tub 295 and 4 1281r. The 5′ end was obtained from a RACE fragment generated using primers B4 756r 5′RACE. *F.hep*-β-tub5: the EST clone 09h06 plasmid insert gave the full sequence of this isotype. *F.hep*-β-tub6: EST clone 18a05 contained from coding nucleotides 302–1286. The sequence was completed using 5′ and 3′RACE fragments generated using primers β-tub6 714r 5′RACE and β-tub6 506f 3′RACE, respectively.

**Fig. 2:**
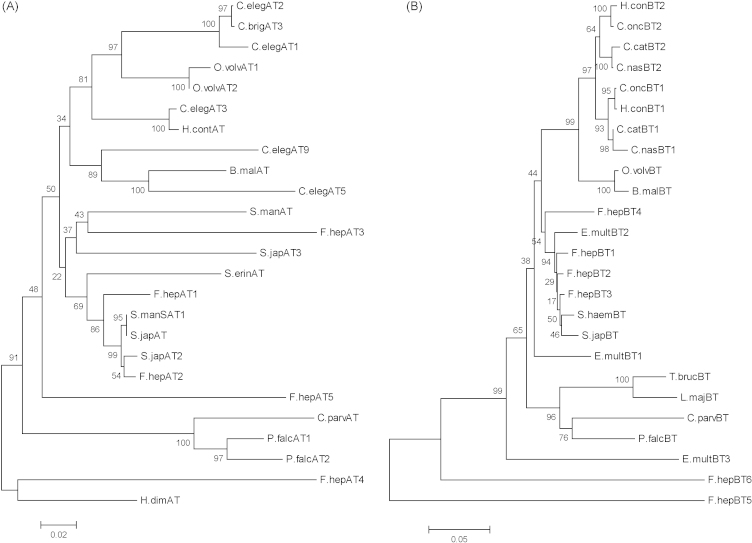
Phylogenetic analyses of (A) Fasciola α-tubulin and (B) β-tubulin isotypes and those of selected nematode, cestode and protozoan species accessed from the GenBank protein database. The Neighbour–Joining method was used and the trees were drawn using MEGA 4.0. All positions containing gaps and missing data were eliminated from the datasets. Numbers on branches represent bootstrapping values as a percentage of 500 replicates. Databank accession numbers are in brackets. (A) α-tubulin isotypes. F.hepAT1 to F.hepAT5: *Fasciola hepatica* α-tubulin isotypes *F.hep*-α-tub1 to *F.hep*-α-tub5 (GenBank accession nos. AM933580–AM933584; S.manSat1: *Schistosoma mansoni* α-tubulin (GenBank accession no. AAA29918); S.manAT: *S. mansoni* α-tubulin (GenBank accession no. AAB21180); S.japAT1: *Schistosoma japonicum* α-tubulin (GenBank accession no. AY815746); S.japAT2: *S. japonicum* α-tubulin (GenBank accession no. AAW27478); S.japAT3: *S. japonicum* α-tubulin (GenBank accession no. AAW26679); H.dimAT: *Hymenolepis diminuta* (GenBank accession no. AAL84895); S.erinAT: *Spirometra erinaceieuropaei* α-tubulin (GenBank accession no. BAA89488); B.malAT: *Brugia malayi* α-tubulin (GenBank accession no. EDP37264); C.brigAT: *Caenorhabditis briggsae* hypothetical protein CBG20310 (GenBank accession no. CAE72973); C.elegAT3: *Caenorhabditis elegans* α-tubulin3 (GenBank accession no. BAA22203); C.elegAT5: *C. elegans* tba5 (GenBank accession no. Q19490*)*; C.elegAT9: *C. elegans* α-tubulin9 (GenBank accession no. Q20221); H.contAT1: *Haemonchus contortus* α-tubulin isotype (GenBank accession no. AAA29167); O.volvAT1: *Onchocerca volvulus* α-tubulin (GenBank accession no. AAX24133); O.volvAT2: *O. volvulus* α-tubulin (GenBank accession no. AAY27745); C.parvAT: *Cryptosporidium parvum* α-tubulin (GenBank accession no. AAD20239); P.falcAT1: *Plasmodium falciparum* α-tubulin (GenBank accession no. CAA34101); P.falcAT2: *P. falciparum* α-tubulin II (GenBank accession no. M34390). (B) β-tubulin isotypes. F.hepBT1 to F.hepBT6: *F. hepatica i*sotypes *F.hep-*β*-*tub1 to *F.hep-*β*-*tub6 (AM933585–AM933590); S.haemBT: *Schistosoma haematobium* β-tubulin (GenBank accession no. AAW66672); S.japBT: *S. japonicum* β-tubulin (GenBank accession no. AAO59417); E.multBT1 to E.multBT3: *Echinococcus multilocularis* β-tubulin isotypes, 1 (GenBank accession no. CAB91640), 2 (GenBank accession no CAB91641) and 3 (GenBank accession no. CAB91642); B.malBT: *B. malayi* β-tubulin (GenBank accession no. AAU12501); C.catBT1: *Cyathostomum catinatum* β-tubulin1 (GenBank accession no. AAM95346); C.catBT2: *C. catinatum* β-tubulin2 (GenBank accession no. ABW90092); CnasBT1: *Cylicocyclus nassatus* β-tubulin1 (GenBank accession no. AAG13959); C.nasBT2: *C. nassatus* β-tubulin2 (GenBank accession no. AAT76619); C.oncBT1: *Cooperia oncophora* β-tubulin1 (GenBank accession no. AAP20434); C.oncBT2: *C. oncophora* β-tubulin2 (GenBank accession no. AAP20435); H.contBT1: *H. contortus* β-tubulin (Tub 8–9) (GenBank accession no. AAA29170); H.contBT2: *H. contortus* β-tubulin (Tub 12–16) (GenBank accession no. AAA29168); O.volvBT: *O. volvulus* β-tubulin (GenBank accession no. AAC13548); C.parvBT: *C. parvum* β-tubulin (GenBank accession no. AAN78304); L.majBT: *Leishmania major* β-tubulin (GenBank accession no. CAA63780); P.falcBT: *P. falciparum* β-tubulin (GenBank accession no. M31205); T.brucBT: *Trypanosoma brucei* (GenBank accession no. XP_001218933).

**Table 1:** Primers used for the determination of tubulin isotype sequences

α-tubulin primers	
α-tub 44	5′ AAATGGGAAATGCTTGYT 3′
α-tub 637	5′ AAATATCATAAATGGCCTCGTT 3′
α-tub 397	5′ CARGGWTTYCTRATWTTCCAYTC 3′
α-tub 1160	5′ GCTTCRGCNATNGCSCTNGT 3′
A1 490r 5′RACE	5′ GCTCAACCACGGCAGTGGAGATTTG 3′
A4 718r 5′RACE	5′ CGGCCGTGAGGGTACTGATCACTTG 3′
A4 543f 3′RACE	5′ CGGTTCCGGATTCACAGCACTACTG 3′

β-tubulin primers	
β-tub 295f^a^	5′AAYAAYTGGGCYAARGGNCAYTA3′
β-tub 1230r a	5′ TCRGTRAAYTCCATYTCRTCCAT 3′
β-tub 1281r	5′CTCCTGGTATTGCTGATATT
B2 737r 5′RACE	5′ AGCTGACCAGGGAAACGCAACAGG 3′
B2 712f 3′RACE	5′ ACCTGTTTGCGTTTCCCTGGTCAGC 3′
B3 519r 5′RACE	5′ GGTCAGCTTCAAAGTGCGGAAGCAA 3′
B3 361f 3′RACE	5′ CTCCGTGGTTCCCTCACCCAAGGTA 3′
B4 756r 5′RACE	5′ TTCCGGAGATCGGCGTTCAGTTGAC 3′
B6 714r 5′RACE	5′ GTGGTCACACCGGACATTGTTGCACT 3′
B6 506f 3′RACE	5′ GCGTATCCGTCGCCCAAAGTATCTGA 3′

Anchored primers used to amplify isotype coding sequences	
α-tub1Fcomp	5′GACGACGACAAGATGCGCGAATGCATCAGTATTC3′
α-tub1Rcomp	5′GAGGAGAAGCCCGGTTTAATATTCTTCACCCAATTCCTGCCC3′
α-tub2Fcomp	5′GACGACGACAAGATGCGCGAATGTATCAGTGTTC3′
α-tub2Rcomp	5′GAGGAGAAGCCCGGTTTAGTATTCTTCACCCTCGCCTTC3′
α-tub3Fcomp	5′GACGACGACAAGATGCGTGAATGCATCAGCGTAC3′
α-tub3Rcomp	5′GAGGAGAAGCCCGGTTTAGTATTCCGCCTCTTCTTCCTC3′
α-tub4Fcomp	5′GACGACGACAAGATGAGGGAATGTATCAGTCTGC3′
α-tub4Rcomp	5′GAGGAGAAGCCCGGTTCAGTATTCCACTTTTTCGTCCAAAATG3′
α-tub5Fcomp	5′GACGACGACAAGATGACCAATAATCATTCACGCG3′
α-tub5Rcomp	5′GAGGAGAAGCCCGGTTCAGTCTAACTCCTTATCATCC3′
β-tub1Fcomp	5′GACGACGACAAGATGCGGGAAATAGTTCACC3′
β-tub1Rcomp	5′GAGGAGAAGCCCGGTTTAGACTTGTTCTCCTTCC3′
β-tub2Fcomp	5′GACGACGACAAGATGCGTGAAATCGTTCATATTC3′
β-tub2Rcomp	5′GAGGAGAAGCCCGGTTTAGGCTTCCTCAGCCTCTTCATC3′
β-tub3Fcomp	5′GACGACGACAAGATGCGTGAGATTGTCCACATTC3′
β-tub3Rcomp	5′GAGGAGAAGCCCGGTCTACGCTTCCTCATTCTCTTC3′
β-tub4Fcomp	5′GACGACGACAAGATGCGGGAAATCGTGCATATG3′
β-tub4Rcomp	5′GAGGAGAAGCCCGGTTTAAGCCTCCTCTTCCCCTTCCTC3′
β-tub5Fcomp	5′GACGACGACAAGATGCGGGAAATTGTTTTTCTACAAG3′
β-tub5Rcomp	5′GAGGAGAAGCCCGGTTCAAATATCAACCTTTGCGTCTTG3′
β-tub6Fcomp	5′GACGACGACAAGATGCGTGAAATTATCTACATTCAG3′
β-tub6Rcomp	5′GAGGAGAAGCCCGGTCTAATGTTCAACATCTTGAAATTG3′

a Primers and PCR conditions taken from [13]. Y: pyrimidine; R: purine; S: C/G; W: A/T; N: any nucleotide.
